# Is investigation of patients with haemoptysis and normal chest radiograph justified?

**DOI:** 10.1136/thx.2008.108795

**Published:** 2009-05-19

**Authors:** M Thirumaran, R Sundar, I M Sutcliffe, D C Currie

**Affiliations:** Dewsbury and District Hospital, Mid Yorkshire Hospitals NHS Trust, Dewsbury, West Yorkshire, UK

## Abstract

**Background:**

Haemoptysis is a common clinical symptom. A small proportion of patients present with haemoptysis and normal chest radiograph. The investigation strategy for this group of patients is unclear. The aim of this study is to see whether further investigations for this group of patients are justified.

**Methods:**

A retrospective analysis was conducted of consecutive patients presenting with haemoptysis and normal chest radiograph over a period of 56 months irrespective of their smoking status. These patients were investigated by CT of the thorax and fibreoptic bronchoscopy.

**Results:**

275 episodes of haemoptysis with normal chest radiograph were investigated further in 270 patients (60% males). The median age was 60 years. Twenty-six patients were diagnosed to have respiratory tract malignancies (larynx, 1; trachea, 1; lung, 22; carcinoid, 1; and leiomyoma, 1). Eight (31%) of the 26 patients with respiratory tract malignancy had radical treatment. Fibreoptic bronchoscopy was diagnostic of cancer in 14 (54%) of the 26 patients with malignancy. CT of the thorax was suggestive of cancer in 24 (96%) of the 25 patients with malignancy.

**Conclusion:**

It is concluded that further investigation of haemoptysis in smokers is justified regardless of the amount or frequency of haemoptysis based on a 9.6% rate of malignancy in this consecutive series. It is recommended that these patients are investigated with CT of the thorax followed by fibreoptic bronchoscopy.

Haemoptysis is a common and worrying clinical symptom. It is potentially life threatening and warrants further investigation. Haemoptysis can be due to varying aetiologies including lung cancer.^1^ The optimal strategy for investigating patients with haemoptysis and normal chest radiograph (CXR) is unclear. Doctors tend to investigate these patients differently.

The prognosis for lung cancer remains poor, with 5-year survival figures of 4–12% worldwide.^2^ ^3^The prospects for changing this are limited by a number of factors including, most notably, the late presentation of patients with advanced disease and the lack of convincing evidence for screening asymptomatic individuals for lung cancer. Early diagnosis of lung cancer is crucial to allow curative resection. Further investigations of patients with haemoptysis and normal CXR may lead to early diagnosis.

In this retrospective study we analysed a group of patients with haemoptysis and normal CXR to see whether further investigations were justified.

## Methods

We conducted a retrospective analysis of consecutive patients presenting with haemoptysis and normal CXR to Dewsbury and District Hospital, West Yorkshire, UK between May 2001 and December 2005. The catchment population of our hospital is 175 000.

All patients presenting with haemoptysis and a normal CXR were investigated further in line with local guidance irrespective of their smoking status, age, or quantity or frequency of haemoptysis. The UK national guidelines^4^ for management of lung cancer recommend urgent CXR for patients presenting with haemoptysis. In our local policy for referral and management of lung cancer for general practitioners (GPs) and general physicians, urgent referral to chest physicians was advised for all patients presenting with haemoptysis regardless of the CXR result. The patients were either referred from primary care locally or from secondary care in our hospital.

The patients were identified from the urgent suspected lung cancer referrals database and our fibreoptic bronchoscopy (FOB) database. Patients with abnormal CXR were excluded. The CXR should have been reported normal by a radiologist or documented to be normal in medical notes by a chest physician. The outpatient CXRs are reported by our radiologists as a standard practice.

These patients were routinely investigated further by CT of the thorax, FOB as well as history, clinical examination and blood investigations. The standard CT procedure in our institution for lung cancer staging during the study period was contrast-enhanced contiguous 5 mm sections from lung apices to the liver and adrenal glands. FOB is performed either by the chest physician or by supervised trainees. From the clinical records the following variables were collected: age, sex, smoking history, comorbidities, symptoms (eg, chest pain, weight loss), quantity/nature (streak, teaspoonful, tablespoonful), frequency and duration of haemoptysis (once only, recurrent lasting up to or more than 1 week), history of chronic sputum production when available and the final diagnosis based on clinical, radiology and histology findings.

## Results

A total of 275 episodes of haemoptysis with normal CXR were investigated further in 270 patients (60% males). The median age was 60 years (interquartile range 22 years); 10% were under the age of 40. Ninety percent of these patients were either active smokers (156) or ex smokers (90). The details of the nature and frequency of haemoptysis are as listed in table 1. The details of associated symptoms and comorbidities are listed in table 2. FOB was performed in 269 patients (98%). CT was performed in 257 patients (94%). The results of FOB and CT are cross-tabulated in table 3. Diagnoses of respiratory tract neoplasias and other diagnoses achieved are as listed in tables 4 and 5, respectively.

**Table 1:** Nature and frequency of haemoptysis in patients

Nature of haemoptysis	No of patients (percentage)	Frequency of haemoptysis			
		Once only	Less than a week	One week or more	Not known
Streaks	165 (60%)	54	85	23	3
Teaspoonful	52 (18.9%)	10	33	8	1
Tablespoonful	39 (14.2%)	10	24	4	1
Egg cup full	1 (0.4)	1	0	0	1
Not specified	18 (6.5%)	8	2	5	3

**Table 2:** Associated symptoms and co-morbidities in patients

	No of patients (percentage)
**Symptoms**	
Weight loss	11 (4%)
Chest pain	11 (4%)
Cough	195 (71%)
Purulent sputum	41 (15%)
**Comorbidities**	
Ischaemic heart disease	30 (10.9%)
Chronic obstructive pulmonary disease	32 (11.6)
Hypertension	15 (5.4%)
Asthma	12 (4.3%)
Diabetes	9 (3.2%)

**Table 3:** Cross-tabulation of CT and bronchoscopy results in patients

	CT scan results			
	Normal	?Malignancy	Benign changes	Not done
Bronchoscopy results				
Normal	158	11*	49	15
?Malignancy†	0	13	1	0
?Malignancy‡	4	6	3	1
Benign changes	4	0	4	0
Not done	3	1§	1	0

Data are presented as numbers. Bronchoscopy, n = 269; CT, n = 257.

*Final diagnosis of malignancy made in 10 patients.

†Confirmed to be malignancy in bronchoscopy samples.

‡Benign pathology diagnosed from bronchoscopy samples.

§Clinical diagnosis of lung malignancy.

**Table 4:** Respiratory tract neoplasias diagnosed

Non-small cell carcinoma	20
Small cell carcinoma	2
Laryngeal carcinoma	1
Tracheal paraganglioma	1
Carcinoid	1
Leiomyoma	1

Data are presented as numbers (n = 26)

**Table 5:** Final diagnosis for each episode of haemoptysis

Acute bronchitis	174
Respiratory tract malignancies	26
Bronchiectasis	20
Pneumonia	16
Unexplained	16
Epistaxis	5
Tuberculosis	4
Pulmonary fibrosis	2
Organising pneumonia	2
Squamous metaplasia	2
Lymphoma	1
Carcinoma of the thyroid	1
Chondroma	1
Hamartoma	1
Pulmonary emboli	1
Sarcoidosis	1
Suture granuloma	1
Aspergilloma	1

Data are presented as numbers (n = 275).

Overall, 26 (9.6%) patients were diagnosed to have respiratory tract malignancy. Nine (35%) of the 26 patients with repiratory tract malignancy were suitable for radical treatment. Eight (31%) of the 26 patients with respiratory tract malignancy had radical treatment. One patient with T2N0M0 non-small cell lung cancer refused radical treatment. Six patients had curative surgical resection (lung, 5; trachea, 1), one had radical radiotherapy and one had photodynamic therapy (PDT). Four other patients underwent surgical resection for probable malignancy, but the final histology showed suture granuloma (1), aspergilloma (1) and organising pneumonia (2). Two patients were diagnosed to have papillary carcinoma of the thyroid and lymphoma incidentally. One patient required embolisation for life-threatening haemoptysis of unknown cause.

Nineteen patients were diagnosed to have malignancy in the 28 patients with bronchoscopy findings suspicious of malignancy (lung, 17; trachea, 1; and larynx, 1). Follow-up bronchoscopies were required in one patient and lung cancer was diagnosed only on the third occasion. Two patients with squamous metaplasia had serial autofluorescence bronchoscopies which ruled out malignancy. FOB was diagnostic of cancer in 14 (54%) of the 26 patients with respiratory tract malignancy (one patient declined FOB).

Twenty-four cancers were diagnosed in the 30 patients who had CT findings suspicious of cancer. Among the 57 patients with abnormal CT scan not suggestive of malignancy, 30 did show unsuspected pulmonary pathology to explain the haemoptysis (bronchiectasis, 16; pneumonia, 11; leiomyoma, 1; pulmonary embolism and bronchiectasis, 1; tuberculosis, 1). The patient with laryngeal carcinoma did not have a CT due to the suspicion of laryngeal cancer at bronchoscopy which was later confirmed by the Ear, Nose and Throat (ENT) surgeons. CT was suggestive of cancer in 24 (96%) of the 25 patients with respiratory tract malignancy (the patient with laryngeal cancer was referred to the ENT team after bronchoscopy without CT). Investigations carried out following CT and FOB in 16 patients (20 procedures) are listed in table 6.

**Table 6:** Investigations following CT and fibreoptic bronchoscopy

CT-guided biopsy	1
Follow-up CT	10
Follow-up bronchoscopy	4
Autofluorescence bronchoscopy	2
Video-assisted thoracoscopy	1
Mediastinoscopy	2

Data are presented as numbers (n = 20).

Among the 26 patients diagnosed to have respiratory tract malignancy in our study, 22 were males (85%) and smokers with an average of 38 pack-years. The median age of these patients was 69 years with age range 33–84 years. Thirteen of these patients reported only streak(s) of haemoptysis. Thirteen patients had only one or two episodes of haemoptysis in total prior to presentation. Seventeen of these patients had haemoptysis lasting less than a week. Only 5 of the 26 patients had recurrent haemoptysis of at least a tablespoonful.

## Discussion

In this retrospective study 9.6% of patients were diagnosed to have respiratory tract malignancy. In spite of our local policy, all the patients who presented with haemoptysis may not have been referred to us, leading to selection bias. Previous studies found a 0–16% cancer detection rate in patients presenting with haemoptysis and normal CXR.^5^ ^6^ ^7^ ^8^ ^9^ ^10^

Haemoptysis is the presenting symptom in up to 19% of the patients with lung cancer.^11^ Poe *et al* found that coughing up >30 ml of blood increased the diagnostic yield.^12^ In our study only 5 patients with lung cancer had a tablespoonful, recurrent haemoptysis, and more than half of the patients had only streak(s) with infrequent episodes of haemoptysis lasting less than a week. Fidan *et al* reported in a retrospective analysis of patients presenting with haemoptysis that just over 40% of patients with lung cancer presented with mild and non-recurrent haemoptysis.^13^

Some studies looked at whether patients above a particular age should be investigated. Sood *et al* analysed all the studies published so far and concluded that age above 50, male sex and smokers of 40 pack-years had the highest predictive value for bronchogenic carcinoma.^14^ In contrast, Cortese *et al* reported radiologically occult lung cancer in 5.5% of patients who were <50 years old.^15^ A primary care cohort study reported higher positive predictive value for cancer in patients aged above 55 years when they present with haemoptysis irrespective of CXR appearance.^16^ The median age of patients with lung cancer in our study is 69 years with male predominance (85%).

Both FOB and CT have definite important roles in evaluating haemoptysis in patients with abnormal CXR for bronchogenic carcinoma. A lung cancer detection rate of up to 21% with bronchoscopy alone in patients with haemoptysis and normal CXR has been reported.^17^ ^18^ ^19^ ^20^ Prospective studies by Tak *et al* and Haro *et al* indicate that FOB and CT are complementary, irrespective of CXR findings.^6^ ^21^ ^22^ Even though FOB is a relatively straightforward procedure it still carries a small risk.

In a long-term outcome study of patients with haemoptysis of unknown aetiology Herth *et al* reported that 6% of patients developed lung cancer over a mean follow-up period of 6.6 years.^23^ Santiago *et al* reported 4% of patients developing lung cancer over a 6-year period when the initial investigations were inconclusive.^24^ All these patients were smokers and >40 years of age. In our study, one patient developed lung cancer after a follow-up period of 20 months. The mean follow-up period in our study population was 36 months.

As far as we are aware, this study reports the largest group of consecutive patients with haemoptysis and normal CXR who were investigated with CT and FOB. Our study demonstrates the importance of investigating such patients. However, our study is retrospective and the characteristics of the patient population prevent us from making recommendations with respect to non-smokers, females or younger people.

## Conclusion

Haemoptysis is a valuable diagnostic clue to resectable lung cancer. More frequently haemoptysis is likely to be secondary to benign pulmonary pathology. Bronchoscopy is invasive; radiation is a safety concern of CT, particularly in young people. CT has the advantage of being more sensitive in diagnosing distant bronchial and parenchymal abnormalities, but FOB is better in evaluating mucosal abnormalities and providing material for pathological diagnosis. CT or bronchoscopy could fail to detect abnormalities when the other modality did, as seen in this study. Even though CT and bronchoscopy are complementary, CT is more likely to detect abnormalities to explain the cause of haemoptysis.

We conclude that further investigation of haemoptysis in smokers with a normal CXR is justified regardless of the amount or frequency of haemoptysis based on a 9.6% rate of malignancy in our consecutive series. We recommend that these patients are investigated with CT followed by bronchoscopy.
